# Cardiovascular Disease-Risk Markers in HIV Patients

**DOI:** 10.4172/2155-6113.1000317

**Published:** 2014-06-12

**Authors:** Bela F. Asztalos, Robert Matera, Katalin V. Horvath, Michael Horan, Mariko Tani, Joseph F. Polak, Sally Skinner, Christine A. Wanke

**Affiliations:** 1 Lipid Metabolism Laboratory, Human Nutrition Research Center on Aging at Tufts University, 711 Washington Street, Boston, MA, USA; 2 Division of Nutrition and Infection, Department of Public Health and Community Medicine, Tufts University School of Medicine, 150 Harrison Avenue, Boston, MA, USA; 3 Department of Radiology, Tufts University School of Medicine, 145 Harrison Avenue, Boston, MA, USA

**Keywords:** HIV, CVD risk, ART

## Abstract

**Objectives:**

HIV-positive patients have an increased risk for CVD; however, the underlying mechanisms are not well understood. Our goal was to assess traditional and emerging CVD-risk factors in the CARE Study, a well-described cohort of HIV-infected adults.

**Methods:**

We analyzed demographic and clinical (viral load, CD4 count, ART regimen, cIMT) data including markers of lipid and glucose homeostasis in 176 HIV-positive subjects receiving regular care for HIV infection.

**Results:**

No significant association between cIMT and LDL-C level was observed. HIV patients had significantly lower level of the large α-1 HDL particles and about 3-fold higher level of the small pre β-1 HDL particles than the normal population, but these parameters were not significantly associated with cIMT. Components of the metabolic syndrome, high TG/low HDL-C, insulin resistance and high BMI, as well as viral load were significant but moderate contributors to increased cIMT.

**Conclusion:**

The major lipid disorder was low HDL-C and high TG level in this HIV-positive cohort. LDL-C was not elevated. These and previously published data indicate that HIV infection and HIV medications influence CVD risk by impairing cholesterol removal (efflux) via ABCA1 from macrophages. Decreasing CVD risk in HIV patients, with impaired cholesterol efflux from macrophages, may require a lower LDL-C goal than recommended for HIV-negative patients and also a better control of TG level.

## Introduction

The effects of human immunodeficiency-virus (HIV) infection and antiretroviral therapy (ART) on serum lipid levels and insulin resistance have been established. Combination ART (cART), whether containing protease inhibitors (PI), or non-nucleoside reverse transcriptase inhibitors (NNRTI) with nucleoside reverse transcriptase inhibitors (NRTI), may have drug-dependent effects on glucose and lipid homeostasis. In addition, HIV-mediated comorbidities co-infections, intestinal or liver dysfunction and lipodystrophy with its regional accumulations of body fat- may also significantly influence lipid and glucose metabolism. Often these confounders exist simultaneously; therefore, an effect of a single factor is difficult to determine.

Studies reporting on the effects of HIV infection on lipid and glucose homeostasis in ART-naïve cohorts showed that in less-advanced HIV disease, HIV RNA levels were inversely associated with serum low-density lipoprotein cholesterol (LDL-C) and high-density lipoprotein cholesterol (HDL-C), but were positively associated with serum triglyceride (TG) levels [1]. It is documented that apolipoprotein (apo) B-containing lipoproteins (very low-density lipoprotein [VLDL], intermediate-density lipoprotein (IDL) and LDL) have a decreased fractional catabolic rate in HIV-positive patients [2]. In advanced HIV disease, co-infections e.g. frequent hepatitis C infection- may exacerbate abnormalities in lipid and glucose metabolism due to damage in the liver and pancreas.

HIV infection has been shown to increase the risk for cardiovascular disease (CVD) in several large studies [3,4]. However, there is a controversy about which risk factors cause CVD complications in patients with HIV. The Infectious Diseases Society of America and the AIDS Clinical Trial Group recommend using the National Cholesterol Education Program Adult Treatment Panel III (NCEP III) guidelines for evaluating and managing dyslipidemia in HIV patients [5]. Traditional CVD-risk factors are: age, gender, family history of CVD, hypertension, diabetes, smoking, elevated LDL-C and low HDL-C levels [6]. However, CVD events in HIV-positive patients are largely independent of these traditional risk factors. Our group has shown that age, smoking, diabetes, insulin, glucose, waist circumference, body mass index (BMI) and TG > 150 to have an impact on CVD surrogate markers [7,8]. Data from the Kaiser Permanente Medical Care Program of Northern California, California Medicaid and the Partners Health Care System in Boston indicated significantly more CVD complications among HIV-positive patients compared to HIV-negative subjects with similar age and gender [9,10]. Moreover, Freiberg *et al*. showed significantly increased risk for acute myocardial infarction (MI) in HIV-positive patients compared to non-infected subjects [11].

The aim of this study was to assess traditional and emerging CVD-risk factors in the CARE Study, a well-described longitudinal cohort of HIV-infected adults.

## Methods

### Study Population

We analyzed data on 176 HIV-positive men and women from an ongoing cardiovascular sub-study (CARE), of a longitudinal study (Nutrition for Healthy Living), which examined nutritional and metabolic concerns in HIV-infected individuals at 6-month intervals. Details of this study have been reported elsewhere [12]. Recruitment for the CARE study began in 2000 and required that participants had been part of NFHL. All patients provided informed consent for the NFHL and CARE studies; both were approved by the Tufts Medical Center Institutional Review Board.

### Patient demographic and clinical characteristics

Demographic data were obtained from interviewer-administered questionnaires. Systolic and diastolic blood pressures (BPs) were assessed with a digital automatic BP monitor. BMI was calculated as weight (in kilograms) divided by the square of height (in meter). Highly active antiretroviral therapy (HAART) was defined as the use of ≥ 3 drugs, with ≥ 1 PI or NNRTI. Carotid Intima Media Thickness (cIMT) and coronary artery calcium score by computed tomography (CAC) were performed at baseline. Demographic information, medical history, BP, body composition, and laboratory values were obtained within 3 months after the carotid ultrasonography and CT [13].

### Laboratory measurements

Levels of plasma total cholesterol, triglycerides, and HDL-C were determined using standard enzymatic methods with reagents from Roche Diagnostics (Indianapolis, IN). Triglycerides were measured in apoB-depleted plasma as well (HDL-TG). LDL-C levels were calculated by the Friedewald formula or, in hypertriglyceridemic samples, were measured directly using Roche kits. Remnant lipoprotein cholesterol (RLP-C) was measured by an immunoseparation technique using kits from Kyowa Medex (Tokyo, Japan) [14]. Non-esterified fatty acids (NEFA) were measured enzymatically (Wako Diagnostics, Richmond, VA).

Apolipoproteins A-I, A-II, B, and E and lipoprotein (a) [Lp (a)] were measured by immunoturbidimetric assays (Wako). ApoA-I-containing HDL subpopulations were determined by 2-dimentional non-denaturing agarose-polyacrylamide gel electrophoresis, immunoblotting, and image analysis as described [15,16]. This method allows for the separation of HDL into pre-β, α, and pre-α particles-separated by charge-followed by separation by size (pre-β 1–2, α 1–4, and pre-α 1–4). ApoA-I levels (mg/dL) in the individual HDL subpopulations were calculated by multiplying the percent distribution of apoA-I in the subpopulations with total plasma concentration of apoA-I. Cholesterol ester transfer protein (CETP) and lecithin-cholesterol acyltransferase (LCAT) masses were measured by ELISA (Alpco Diagnostics, Salem, NH). CETP and LCAT activities were determined as described by Fielding [17].

C-reactive protein (CRP) was measured using high-sensitivity (hs) kits (Wako). Tumor Necrosis Factor (TNF)-alpha, Interlukin (IL)-6, soluble Intercellular Adhesion Molecule (sICAM) 1-costimulatory protein (CD) 54 levels were measured by ELISA (R&D Systems, MN). CD4+ cell counts were determined by flow cytometry. HIV RNA levels were quantified using Roche Amplicor Version 1.5 (limit of detection, 400 copies/mL). Homeostasis Model Assessment (HOMA) was calculated.

### Measurement of surrogate markers

To ensure standardized methods for c-IMT and its interpretation, we used protocols adapted from the Cardiovascular Health Study [18]. Centrally trained and certified ultrasonographers performed the imaging, and a single reader at a central reading site interpreted the c-IMT. One longitudinal lateral view of the distal 10 mm of the right and left common carotid artery and 3 longitudinal views in different imaging planes (anterior, lateral, and posterior) of the right and left internal carotid artery were obtained. The mean of the maximum of the near-and far-wall c-IMT was used for the final analysis, because it has been shown to have the strongest association with CVD risk [19]. cIMT was used as a continuous measure and stratified as <0.6 mm, <0.8 mm, and <1.0 mm. Quality control analysis of 32 subjects resulted in intra-class correlation coefficients of 0.911, which is consistent with results of other studies using the same technique in non-HIV-infected populations [20]. Based on the average age, ≤ 0.645 mm was selected as normal, which is consistent with measurement using the same technique in non–HIV-infected populations.

### Statistical analysis

This is a cross-sectional analysis of 176 participants. Analyses were performed with SAS for Windows, version 9.0 (SAS Institute). After distribution assumptions were tested, nonnormally distributed variables were transformed by taking the natural logarithm. Comparisons between groups were conducted using Student’s **t** tests and analysis of variance for continuous variables and the χ^2^ test for binary variables. Univariate logistic regression was conducted for all predictor variables. Separate multivariate models were fit using c-IMT as the dependent variables. All predictor variables with **P** < 0.20 in the univariate analyses were included in the initial multivariate models. The final models were determined using stepwise linear regression techniques. **P** < 0.05 was considered to be statistically significant.

## Results

Demographic, clinical and biochemical characteristics of HIV-infected subjects participating in the CARE study are shown in Tables 1a and 1b. Since levels of some parameters associated with CVD-risk are gender-specific, data are presented by gender; though the majority of the study participants were male. There were statistically significant differences between males and females in age, BMI and CD4 count: women were younger but had higher BMI and viral load compared to men. Use of cART was comparable between males and females. CIMT measurements, HOMA and inflammatory markers (hsCRP, TNF-alpha, IL-6, sICAM1-CD54) were similar between the two genders. About 40% of the study population had HOMA > 2.6. Common cIMTs were 0.59 mm and 0.58 mm, respectively, in males and females, with 30% of both groups having abnormal common cIMTs. Total cholesterol and LDL-C levels were comparable between males and females and were not elevated. Male patients had significantly higher plasma TG levels; higher RLP-C levels (ns) and significantly lower NEFA levels than female patients. Females had significantly higher HDL-C, apoA-I and large HDL particles (α-1 and α-2) compared to males; however, preβ-1, α-3 and α-4 levels were comparable. HDL-C levels were low for both sexes (male: 35 mg/dL, female: 48 mg/dL); however HDLs were equally enriched in TGs. There were no significant differences between the two genders in apoA-II and apo E levels; however apoB levels were significantly lower in females compared to males. Moreover, there were no significant differences in the two most important HDL-modifying factors: CETP and LCAT either in concentrations or activities between males and females.

In Tables 2a and 2b, data are presented according to viral load. Patients with undetectable level of viral RNA (viral copies <400) used more ART in any combination than patients with detectable viral RNA. Patients with the highest viral load had higher levels of inflammatory markers (hsCRP, IL-6 and TNF-α) than the other two groups. In case of TNF-α, the difference was significant. There was an inverse association, although not significant, between abnormal cIMT measures and viral load. The group with no detectable viral load had moderately but significantly higher total cholesterol, LDL-C, RLP-C, and LCAT levels than the other two groups. On the other hand, patients with the highest viral level had significantly lower HDL-C and apoA-I levels than the other two groups. The HDL subpopulation profiles were similar in the 3 groups.

In Tables 3a and 3b, data are presented in LDL-C tertiles [lower (median=71 mg/dL), middle (median=106 mg/dL), upper (median=144 mg/dL)]. As expected, LDL-C level had significant positive correlations with apoB and Lp(a) levels. Unexpectedly, LDL-C level had a significant positive association with HDL-C level and a significant inverse association with ICAM-1 level. LDL-C level showed a significant inverse association with viral load, but did not show any association with cIMT.

The most common lipid disorder in HIV-positive patients is marked with low HDL-C and high TG levels. We have selected TG >150 mg/dL and HDL-C< 40 mg/dL as cut points. Patients with high TG and low HDL-C had the highest cIMT measures despite having the lowest LDL-C level (Tables 4a and 4b). The HDL subpopulation profile was in line with the changing HDL-C and TG levels. Low HDL-C/high TG level was associated with very low levels of the large (α-1, α-2, preα-1, and preα-2) HDL particles. The group with low HDL-C and high TG level had very low apoA-I level (111 mg/dl vs. 145 mg/dl in the high HDL-C low TG group), but had the highest apoE level. We were not powered to analyze the effects of HIV medication on CVD risk. However, we observed that plasma TG level had a positive association with use of HIV medication (Table 4a). Moreover, increased plasma TG levels were associated with higher cIMT indiscriminately whether it was caused by HIV medication or else.

When patients were tertiled by BMI (<21 kg/m^2^, 21–30 kg/m^2^, >30 kg/m^2^) (Tables 5a and 5b), BMI was significantly and positively associated with CD4 count, hsCRP level, abnormal HOMA and cIMT measures. However, there were no significant differences in viral load and any of the lipoprotein-associated parameters among the three groups [except for Lp(a)]. We have tested whether impaired glucose metabolism was the underlying mechanism for the higher cIMT measures in the high BMI group (data not shown). Patients with high HOMA had significantly higher cIMT measures than patients with normal HOMA (36% vs.24%); however, the significance was weaker compared to BMI (p=0.036 vs. p=0.003). In addition, the association was confounded with significantly higher TG levels in the high HOMA group [162 mg/dl vs. 109 mg/dl (p=0.005)].

When patents were stratified according to cIMT measures into normal (<0.645 mm) and abnormal (>0.645 mm) groups, we observed significantly higher BMI [28 vs.25 (p<0.001)], HOMA [51% vs.33% (p=0.036)], and TG level [169 mg/dLvs. 159 mg/dL (p=0.045)] in the group with higher cIMT, (data not shown).

## Discussion

It is widely accepted that HIV-positive patients have increased risk for CVD. Grunfeld *et al.* published that HIV-infected individuals had an average of 0.188 mm higher measures of IMT than controls after adjusting for demographic characteristics [21]. The significance level was only slightly attenuated after adjusting for traditional CVD-risk factors.

The underlying mechanism for the increased CVD risk in HIV-infected individuals is elusive. Generally, HIV patients do not have higher LDL-C level than the normal population. However, they have higher prevalence of dyslipidemia, marked with elevated TG and low HDL-C levels. It is well documented that HIV infection itself and certain HIV medications increase TG level [22,23]. The mechanisms are not clear though: decreased clearance of TG-rich lipoproteins was reported both in treated and non-treated HIV-positive subjects [24]. Increased TG level is accompanied with increased CETP activity which in turn decreases HDL-C level. CETP transfers cholesterol esters from HDL to apoB-containing lipoproteins in exchange for TG. HDL becomes enriched in TG and a good substrate for hepatic lipase resulting in the decrease of the large cholesterol-rich α-1 and in the increase of the small lipid-poor preβ-1 HDL particles.

There is another mechanism responsible for decreased HDL-C in HIV-positive patients. HIV, via the viral accessory protein Nef, stimulates the degradation of ATP-binding cassette transporter A1 (ABCA1) and impairs cholesterol efflux from macrophages, causing accumulation of cholesterol and the transformation of macrophages into foam cells, a hallmark of atherosclerosis [25,26]. Extracellular Nef, secreted by HIV-infected cells, can inhibit cholesterol efflux from uninfected cells as well. On the other hand, stimulation of cholesterol efflux through activation of ABCA1 suppresses HIV-1 replication and infectivity [27]. These findings suggest that interaction between Nef and ABCA1 may be essential for both viral replication and impairment of cellular lipid metabolism. ABCA1-mediated cholesterol efflux is essential for the maturation of the lipid-poor preβ-1 particles into larger more lipidated particles with α-1 HDL being the largest [28]. Cholesterol from α-1 particles is transferred to the bile via scavenger receptor B1 (SRB1) [29]. However, this cycle (reverse cholesterol transport) can be disturbed when concentrations of TG-rich lipoproteins (VLDL and RLP) are increased. Increased TG-rich particles stimulate CETP activity. CETP mediates cholesteryl ester exchange for TG between α-1 HDL and TG-rich lipoproteins resulting in decreased cholesterol removal to the bile. The other consequence of CETP activity is that the TG-enriched HDL is a good substrate for hepatic lipase and the removal of the lipid core transforms large α-1 HDL into preβ-1 HDL particles [30]. Preβ-1 level in HIV-positive patients in this study was about 3-fold higher while α-1 level was less than half compared to age- and gender-matched subjects free of CVD and HIV infection selected from the Framingham Offspring Study population [31].

An HDL subpopulation profile, characterized by low α-1 and high preβ-1 levels, seen in HIV-positive subjects, is in line with the hypothesis that these subjects have impaired ABCA1 (decreased) and CETP (increased) functions. These two mechanisms might explain the lack of significant association between cIMT and LDL-C level in this population. Increased LDL-C level is usually associated with cholesterol accumulation in macrophages and increased foam-cell formation. However, impaired cell-cholesterol efflux, as a result of decreased ABCA1 level, helps macrophages cumulate intracellular cholesterol even in the absent of increased LDL-C level.

Recent publications documented that rather impaired HDL functions (decreased cholesterol efflux, anti-inflammatory, and anti-oxidative) than low HDL-C is associated with increased CVD risk in the general population [32,33]. Impaired HDL functions have also been documented in the HIV population [34,35]. The high prevalence of dyslipidemia and significantly altered HDL subpopulation profile make us presume that our patients have dysfunctional HDL.

In conclusion, we have shown that traditional CVD-risk factors-total, LDL and HDL-cholesterol do not reflect the true CVD risk in HIV-positive patients. Components of the metabolic syndrome, high TG/low HDL-C, insulin resistance and high BMI, are significant contributors in carotid thickening. Moreover, both HIV infection and HIV medications influence CVD risk by impairing cholesterol removal from macrophages. Based on our data, we believe that CVD risk calculators, using age, LDL-C, CRP, and family history, do not show the real CVD risk in HIV patients. We assume that decreasing CVD risk in HIV patients, with impaired cholesterol efflux from macrophages, requires a very low LDL-C goal and better control of the TG level.

## Figures and Tables

**Table 1a:** Major characteristics of the subjects divided by gender.

VARIABLE	Males (n=29)	Females n=37	P
**Age**	46 ± 7	43 ± 6	0.024
**BMI**	25 (23, 28)	27 (24, 35)	0.019
**Log10 Viral Load** (copies/mL)	2.3 (2.3, 3.9)	2.3 (2.3, 3.7)	0.96
**Undetectable Viral Load**	54%	51%	0.75
**CD4 (count/cu mm)**	386 (230, 585)	447 (362, 640)	0.027
**ART**	78%	68%	0.18
**NRTIs**	77%	65%	0.15
**NNRTIs**	36%	22%	0.11
**PIs**	44%	43%	0.92
**HAART**	67%	54%	0.13
**PI & NRTI-HAART**	33%	32%	0.92
**NNRTI and NRTI-HAART**	23%	14%	0.20
**PI and NNRTI-HAART**	11%	8%	0.77*
**CRP** (μg/mL)	1.9 (0.7, 3.6)	1.4 (0.6, 3.6)	0.80
**TNF-alpha** (pg/mL)	1.6 (1.1, 2.3)	1.5 (0.9, 2.2)	0.71
**IL-6** (pg/mL)	1.6 (1.1, 2.6)	1.6 (1.3, 2.9)	0.47
**sICAMI-CD54** (ng/dL)	259 (192, 359)	326 (182, 447)	0.11
**HOMA** > 2.6	38%	40%	0.85
**Common cIMT** (mm)	0.590 (0.513, 0.665)	0.583 (0.531, 0.682)	0.78
**Abnormal Common cIMT** (> 0.645 mm)	29%	30%	0.97

Normally distributed parameters are expressed as average ± SD.

Not normally distributed parameters are expressed as median (min, max).

**Table 1b:** Lipoprotein-associated parameters in subjects grouped by gender.

VARIABLE	Male (n=29)	Female (n=37)	P
**LDL-C**	108 ± 36	110 ± 32	0.84
**TG**	144 (83, 226)	103 (85, 131)	0.016
**RLP-C**	9.3 (6.5, 16.6)	7.8 (6.4, 11.0)	0.062
**NEFA**	0.3 (0.2, 0.4)	0.4 (0.3, 0.5)	0.011
**Apo-B**	87 ± 23	79 ± 18	0.037
**LP(a)**	17 (7, 53)	23 (13, 33)	0.36
**HDL-C**	35 (28, 46)	48 (33, 61)	0.004
**HDL-TG**	17 (11, 26)	17 (14, 21)	0.99
**ApoA-I**	122 ± 26	138 ± 32	0.003
**ApoA-II**	35 ± 9	34 ± 11	0.36
**ApoE**	4.0 (3.3, 5.4)	4.4 (3.6, 5.2)	0.41
**Preβ-1**	36 (29, 46)	36 (25, 47)	0.40
**Preβ-2**	1.5 (1.1, 2.9)	1.7 (1.1, 2.8)	0.58
**α-1**	9 (5, 14)	17 (9, 29)	< 0.001
**α-2**	26 (22, 33)	35 (26, 40)	0.002
**α-3**	23 (18, 28)	20 (15, 28)	0.28
**α-4**	7 (5, 10)	7 (6, 10)	0.59
**Preα-1**	2 (1, 4)	4 (2, 11)	0.002
**Preα-2**	3 (2, 5)	4 (3, 8)	0.034
**Preα-3**	3 (2, 4)	3 (2, 4)	0.41
**Preα-4**	1.2 (0.7, 1.6)	1.1 (0.6, 1.8)	0.95
**CETP** (μg/mL)	2 (2, 3)	2 (2, 3)	0.66
**LCAT** (μg/mL)	7 (6, 8)	7 (6, 8)	0.42
**CETP Activity** (μg/mL/hour)	0.3 (−1.3, 1.8)	0.8 (−0.7, 2.0)	0.36
**LCAT Activity** (μg/mL/hour)	17 (10, 27)	13 (8, 30)	0.39

Values are mg/dL or as indicated.

Normally distributed parameters are expressed as average ± SD.

Not normally distributed parameters are expressed as median (min, max).

**Table 2a:** Major characteristics in patients, tertiled by their viral load (VL)

VARIABLE	VL < 400 (n=93)	VL 400–10,000 (n=43)	VL > 10,000 (n=38)	P
**Age** (year)	45 ± 7	45 ± 7	46 ± 7	0.78
**BMI** (kg/m^2^)	26 (24, 29)	25 (23, 28)	25 (23, 28)	0.21
**Log10 Viral Load** (copies/mL)	2.3 (2.3, 2.3)	3.4 (3.0, 3.7)	4.6 (4.3, 5.1)	
**Undetectable Viral Load**				
(<400 copies/mL)	100%	0	0	
**CD4** (count/mm^3^)	469 (305, 633)	392 (248, 585)	276 (137, 423)	< 0.001
**ART**	95%	60%	50%	< 0.001
**NRTIs**	90%	60%	50%	< 0.001
**NNRTIs**	48%	14%	13%	< 0.001
**PIs**	57%	33%	29%	0.002
**HAART**	84%	42%	42%	< 0.001
**PI & NRTI-HAART**	38%	28%	29%	0.43
**NNRTI and NRTI-HAART**	28%	9%	13%	0.022
**PI and NNRTI-HAART**	18%	5%	0	0.002
**hsCRP** (μg/mL)	1.8 (0.7, 3.7)	1.8 (0.7, 3.8)	1.9 (0.8, 3.9)	0.97
**TNF-alpha** (pg/mL)	1.2 (0.9, 1.7)	1.8 (1.3, 2.7)	2.2 (1.5, 2.9)	< 0.001
**IL-6** (pg/mL)	1.5 (1.1, 2.6)	1.7 (1.2, 2.7)	1.8 (1.3, 3.6)	0.31
**sICAM1-CD54** (ng/dL)	255 (172, 359)	309 (186, 403)	275 (219, 381)	0.30
**HOMA > 2.6**	39%	37%	36%	0.93
**CAC (>400)**	3%	5%	0%	0.42
**Common cIMT** (mm)	0.60 (0.51, 0.69)	0.57 (0.50, 0.66)	0.57 (0.53, 0.63)	0.34
**Abnormal Common cIMT** (> 0.645 mm)	34%	28%	19%	0.23

Normally distributed parameters are expressed as average ± SD.

Not normally distributed parameters are expressed as median (min, max).

**Table 2b:** Lipoprotein associated parameters in patients, tertiled by their viral load (VL).

VARIABLE	VL < 400 (n=93)	VL 400–10,000 (n=43)	VL > 10,000 (n=38)	P
**Total-C**	193 (161, 223)	179 (158, 204)	167 (133, 186)	< 0.001
**LDL-C**	112 (84, 136)	107 (87, 131)	96 (69, 119)	0.013
**Triglycerides**	160 (85, 235)	127 (75, 173)	112 (90, 212)	0.29
**RLP-C**	11 (7, 19)	7 (6, 13)	8 (6, 13)	0.005
**NEFA** (mEq/L)	0.4 (0.2, 0.5)	0.3 (0.2, 0.4)	0.3 (0.2, 0.5)	0.076
**Apo-B**	89 ± 24	83 ± 18	78 ± 19	0.057
**Lp(a)**	19 (8, 44)	22 (9, 40)	15 (3, 28)	0.19
**HDL-C**	40 (32, 48)	38 (29, 51)	32 (24, 38)	0.003
**HDL-TG**	17 (12, 24)	19 (11, 25)	17 (12, 24)	0.93
**ApoA-I**	129 ± 28	130 ± 30	111 ± 22	0.002
**ApoA-II**	36 ± 10	35 ± 9	32 ± 10	0.12
**ApoE**	4.5 (3.5, 5.8)	3.6 (3.1, 4.4)	3.9 (3.0, 4.6)	< 0.001
**Preβ-1**	37 (30, 47)	38 (31, 43)	35 (22, 40)	0.17
**Preβ-2**	1.8 (1.1, 3.4)	2.1 (1.1, 2.6)	1.3 (0.9, 2.3)	0.12
**α-1**	10 (6, 17)	11 (7, 17)	9 (6, 12)	0.18
**α-2**	29 (23, 35)	30 (24, 36)	26 (22, 30)	0.25
**α-3**	21 (17, 26)	25 (19, 31)	23 (16, 28)	0.14
**α-4**	7 (5, 10)	7 (6, 10)	7 (5, 9)	0.40
**Preα-1**	2.6 (1.0, 5.0)	2.5 (1.2, 5.2)	1.4 (0.6, 3.4)	0.11
**Preα-2**	4.0 (2.5, 5.5)	3.8 (2.5, 5.5)	3.2 (1.9, 4.4)	0.26
**Preα-3**	2.9 (.12, 4.1)	2.9 (2.0, 4.0)	2.2 (1.8, 4.0)	0.28
**Preα-4**	1.2 (0.6, 1.6)	1.3 (0.8, 1.8)	1.0 (0. 7, 1.3)	0.27
**CETP** (μg/mL)	2.3 (1.9, 2.7)	2.1 (1.7, 2.5)	1.9 (1.6, 2.6)	0.37
**LCAT** (μg/mL)	7.6 (6.6, 8.4)	6.7 (5.8, 7.6)	6.9 (5.3, 7.7)	0.012
**CETP Activity** (μg/mL/hour)	0.59 (−1.42, 1.85)	0.47 (−0.72, 2.44)	0.03 (−0.57, 1.49)	0.93
**LCAT Activity** (μg/mL/hour)	17 (11, 27)	17(10, 27)	16 (8, 25)	0.34

Values are mg/dL or as indicated.

Normal distributed data presented as average ± SD.

Non-normal distributed data presented as median (min. and max)

**Table 3a:** Characteristics of the subjects, tertiled by LDL-C level.

VARIABLE	LDL-C ≤ 92mg/dL (n=59)	LDL-C 93–122 mg/dL (n=59)	LDL-C ≥ 123 mg/dL (n=58)	P
**Age** (year)	45 ± 7	46 ± 7	44 ± 6	0.47
**BMI** (kg/m^2^)	26 (22, 28)	25 (23, 29)	26 (23, 28)	0.87
**Log10 Viral Load** (copies/mL)	2.8 (2.3, 4.2)	2.9 (2.3, 4.0)	2.3 (2.3, 3.0)	0.024
**Undetectable Viral Load** (<400 copies/mL)	28 (48%)	26 (46%)	39 (66%)	0.054
**CD4** (count/mm^3^)	389 (208, 640)	403 (312, 520)	390 (230, 609)	0.90
**ART**	81%	69%	80%	0.23
**NRTIs**	76%	69%	78%	0.48
**NNRTIs**	31%	22%	43%	0.051
**PIs**	45%	45%	45%	1.00
**HAART**	66%	60%	68%	0.66
**PI & NRTI-HAART**	38%	38%	25%	0.23
**NNRTI and NRTI-HAART**	22%	16%	23%	0.52
**PI and NNRTI-HAART**	5%	7%	20%	0.025
**hsCRP** (μg/mL)	1.6 (0.4, 3.6)	1.8 (0.9, 2.8)	1.9 (0.9, 4.2)	0.22
**TNF-alpha** (pg/mL)	1.9 (1.3, 2.6)	1.5 (1.0, 2.2)	1.2 (0.9, 1.7)	0.002
**IL-6** (pg/mL)	2.0 (1.3, 3.0)	1.4 (1.1, 2.7)	1.5 (1.0, 2.5)	0.077
**sICAM1-CD54** (ng/dL)	345 (200, 463)	260 (182, 325)	227 (178, 335)	0.030
**HOMA > 2.6**	46%	33%	34%	0.29
**CAC > 400**	2 (4%)	2 (4%)	1 (2%)	0.87
**Common cIMT** (mm)	0.59 (0.51, 0.66)	0.59 (0.50, 0.68)	0.59 (0.53, 0.66)	0.93
**Abnormal Common cIMT** (> 0.645 mm)	29%	33%	27%	0.74

Normally distributed parameters are expressed as average ± SD.

Not normally distributed parameters are expressed as median (min, max).

**Table 3b:** Lipoprotein-associated parameters in patients, divided by LDL-C level.

VARIABLE	≤ 92mg/dL (n=59)	93–122 mg/dL (n=59)	≥ 123 mg/dL (n=59)	P
**LDL-C**	71 (61, 82)	106 (100, 115)	144 (132, 160)	< 0.001
**Triglycerides**	128 (81, 237)	146 (84, 250)	126 (85, 188)	0.57
**RLP-C**	7.9 (5.4, 18.8)	10.3 (6.6, 18.6)	8.8 (7.1, 14.7)	0.44
**NEFA** (mEq/L)	0.3 (0.3, 0.4)	0.4 (0.2, 0.5)	0.3 (0.2, 0.5)	0.97
**Apo-B**	65 (52, 80)	86 (76, 94)	98 (92, 107)	< 0.001
**Lp(a)**	15 (6, 30)	15 (5, 40)	26 (15, 59)	0.006
**HDL-C**	33 (25, 48)	36 (31, 46)	42 (33, 49)	0.013
**HDL-TG**	18 (14, 25)	18 (11, 28)	14 (10, 19)	0.039
**ApoA-I**	118 (98, 142)	126 (108, 139)	125 (111, 141)	0.29
**ApoA-II**	33 (27, 41)	33 (30, 40)	37 (29, 43)	0.23
**ApoE**	4.0 (3.3, 5.3)	4.3 (3.4, 5.3)	4.2 (3.5, 5.5)	0.76
**Preβ-1**	35 (24, 46)	39 (32, 46)	35 (29, 43)	0.30
**Preβ-2**	1.5 (0.9, 2.8)	1.5 (1.0, 2.6)	2.1 (1.2, 3.5)	0.094
**α-1**	10 (5, 17)	10 (7, 17)	10 (6, 14)	0.86
**α-2**	26 (22, 32)	30 (25, 39)	28 (23, 35)	0.13
**α-3**	22 (17, 28)	23 (18, 27)	23 (18, 30)	0.83
**α-4**	7 (5, 10)	7 (5, 9)	8 (6, 10)	0.94
**Preα-1**	2.3 (0.6, 6.2)	2.6 (0.8, 5.1)	2.5 (1.2, 4.1)	0.93
**Preα-2**	3.4 (2.1, 4.6)	4.1 (2.3, 5.7)	3.8 (2.7, 5.2)	0.50
**Preα-3**	2.9 (2.1, 4.0)	2.8 (1.9, 3.8)	2.8 (2.1, 4.5)	0.68
**Preα-4**	1.2 (0.7, 1.5)	1.2 (0.6, 1.7)	1.1 (0.5, 1.7)	0.82
**CETP** (μg/mL)	2.0 (1.7, 2.5)	2.2 (1.7, 2.6)	2.3 (2.0, 2.8)	0.050
**LCAT** (μg/mL)	7 (6, 8)	7 (6, 8)	7 (6, 9)	0.42
**CETP Activity** (μg/mL/hour)	−0.1 (−1.3, 1.8)	0.9 (−0.3, 2.7)	0.2 (−0.9, 1.8)	0.17
**LCAT Activity** (μg/mL/hour)	17 (11, 26)	18 (11, 27)	14 (8, 27)	0.41

Values are mg/dL or as indicated.

Parameters are expressed as median (min, max).

**Table 4a:** Characteristics of the subjects, quartiled by HDL-C and TG levels.

VARIABLE	HDL ≥ 40 mg/dL TG < 150 mg/dL(n=55)	HDL ≥ 40 mg/dL TG ≥ 150 mg/dL(n=21)	HDL < 40 mg/dL TG < 150 mg/dL(n=42)	HDL < 40 mg/dL TG ≥ 150 mg/dL(n=57)	P
**Age** (year)	45 ± 6	43 ± 6	44 ± 7	47 ± 7	0.079
**BMI** (kg/m^2^)	26 (22, 27)	25 (24, 29)	25 (23, 28)	26 (23, 29)	0.56
**Log10 Viral Load** (copies/mL)	2.3 (2.3, 3.5)	2.3 (2.3, 2.3)	3.7 (2.3, 4.5)	2.3 (2.3, 3.9)	< 0.001
**Undetectable Viral Load** (<400 copies/mL)	57%	80%	31%	56%	0.002
**CD4** (count/mm^3^)	424 (278, 640)	435 (317, 627)	362 (220, 550)	400 (221, 625)	0.22
**ART**	71%	100%	55%	89%	< 0.001
**NRTIs**	67%	95%	52%	89%	< 0.001
**NNRTIs**	35%	48%	19%	33%	0.12
**PIs**	36%	67%	26%	58%	0.001
**HAART**	55%	95%	38%	82%	< 0.001
**PI & NRTI-HAART**	24%	48%	19%	49%	0.002
**NNRTI and NRTI-HAART**	18%	29%	14%	25%	0.46
**PI and NNRTI-HAART**	13%	19%	5%	9%	0.29
**hsCRP** (μg/mL)	1.9 (0.7, 3.6)	1.6 (0.7, 2.4)	1.5 (0.5, 4.0)	2.2 (0.9, 3.9)	0.58
**TNF-alpha** (pg/mL)	1.5 (1.1, 2.3)	1.2 (0.9, 1.7)	1.8 (1.3, 2.9)	1.6 (1.0, 2.0)	0.11
**IL-6 (pg/mL)**	1.7 (1.1, 2.3)	1.5 (0.8, 1.8)	1.6 (1.2, 3.0)	1.6 (1.1, 2.8)	0.50
**sICAM1-CD54** (ng/dL)	259 (179, 359)	209 (165, 376)	291 (203, 434)	262 (208, 376)	0.33
**HOMA > 2.6**	26%	53%	35%	47%	0.076
**CAC > 400**	2%	5%	3%	4%	0.74
**Common cIMT** (mm)	0.58 (0.52, 0.65)	0.60 (0.51, 0.66)	0.57 (0.50, 0.63)	0.62 (0.54, 0.80)	0.098
**Abnormal Common cIMT** (> 0.645 mm)	26%	26%	22%	41%	0.18

Normally distributed parameters are expressed as average ± SD.

Not normally distributed parameters are expressed as median (min, max).

**Table 4b:** Lipoprotein-associated parameters in patients, quartiled by HDL-C and TG levels.

VARIABLE	HDL ≥ 40 mg/dL TG < 150 mg/dL (n=55)	HDL ≥ 40 mg/dL TG ≥ 150 mg/dL (n=21)	HDL < 40 mg/dL TG < 150 mg/dL (n=42)	HDL < 40 mg/dL TG ≥ 150 mg/dL (n=57)	P
**LDL-C**	117 (84, 143)	120 (106, 152)	103 (78, 127)	94 (67, 119)	< 0.001
**Triglycerides**	83 (52, 111)	188 (179, 226)	92 (70, 111)	254 (186, 371)	< 0.001
**RLP-C**	7 (6, 8)	12 (9, 18)	6 (5, 8)	18 (12, 26)	< 0.001
**NEFA** (mEq/L)	0.4 (0.2, 0.5)	0.3 (0.2, 0.4)	0.3 (0.2, 0.4)	0.3 (0.2, 0.4)	0.66
**Apo-B**	78 ± 18	99 ± 16	75 ± 17	93 ± 20	< 0.001
**Lp(a)**	23 (14, 48)	15 (7, 53)	17 (4, 41)	14 (7, 34)	0.11
**HDL-C**	51 (46, 62)	44 (42, 50)	32 (27, 35)	29 (25, 34)	< 0.001
**HDL-TG**	14 (11, 19)	26 (16, 30)	13 (9, 17)	20 (17, 29)	< 0.001
**ApoA-I**	145 ± 24	148 ± 24	106 ± 16	111 ± 18	< 0.001
**ApoA-II**	37 ± 9	45 ± 9	30 ± 9	33 ± 8	< 0.001
**ApoE**	3.8 (3.5, 4.6)	4.5 (4.0, 5.8)	3.5 (2.9, 3.9)	5.4 (4.3, 7.2)	< 0.001
**Preβ-1**	37 (29, 47)	43 (33, 47)	30 (22, 36)	39 (31, 46)	0.001
**Preβ-2**	2.6 (1.3, 3.7)	1.7 (1.2, 2.2)	1.3 (0.9, 2.1)	1.2 (0.9, 2.3)	< 0.001
**α-1**	18.9 (14.9, 29.3)	10.9 (8.1, 12.1)	8.8 (6.7, 11.8)	5.8 (3.5, 8.9)	< 0.001
**α-2**	35 (28, 41)	36 (33, 38)	26 (21, 30)	24 (19, 27)	< 0.001
**α-3**	22 (17, 29)	30 (24, 33)	23 (16, 26)	20 (17, 26)	0.006
**α-4**	6.9 (5.9, 10.5)	9.5 (7.7, 11.4)	7.3 (6.1, 9.3)	6.2 (4.7, 8.1)	0.013
**Preα-1**	5.0 (2.5, 9.9)	2.6 (0.6, 3.9)	1.9 (0.6, 3.6)	1.3 (0.3, 2.5)	< 0.001
**Preα-2**	5.0 (2.9, 8.2)	3.4 (2.5, 6.5)	3.5 (2.1, 4.6)	3.2 (2.0, 4.3)	0.002
**Preα-3**	2.8 (1.9, 4.3)	3.5 (2.1, 5.4)	2.4 (1.8, 3.5)	2.7 (1.9, 3.4)	0.28
**Preα-4**	1.2 (0.5, 1.9)	1.5 (0.7, 2.1)	0.9 (0.6, 1.4)	1.2 (0.7, 1.5)	0.17
**CETP** (μg/mL)	2.2 (1.8, 2.5)	2.2 (1.6, 2.4)	2.2 (1.8, 2.7)	2.1 (1.7, 2.6)	0.80
**LCAT** (μg/mL)	6.9 (5.7, 7.7)	8.2 (7.4, 10.1)	5.9 (5.3, 6.9)	7.7 (6.8, 8.7)	< 0.001
**CETP Activity** (μg/mL/hour)	−0.41 (−1.54, 1.01)	0.33 (−0.85, 2.44)	0.02 (−1.21, 0.87)	1.81 (0.35, 2.98)	0.001
**LCAT Activity** (μg/mL/hour)	13 (7, 26)	16 (12, 24)	12 (7, 23)	20 (13, 28)	0.014

Values are mg/dL or as indicated.

Normally distributed parameters are expressed as average ± SD.

Not normally distributed parameters are expressed as median (min, max).

**Table 5a:** Characteristics of the patients, tertiled by BMI.

VARIABLE	BMI < 21 (n=20)	BMI 21–30 (n=128)	BMI > 30 (n=28)	P
**Age** (year)	47 ± 8	45 ± 7	43 ± 7	0.30
**BMI** (kg/m^2^)	21 (20, 21)	25 (23, 27)	34 (31, 36)	
**Log10 Viral Load** (copies/mL)	2.3 (2.3, 4.0)	2.3 (2.3, 3.9)	2.3 (2.3, 3.5)	0.73
**Undetectable Viral Load** (<400 copies/mL)	50%	53%	57%	0.88
**CD4** (count/mm^3^)	328 (193, 392)	396 (235, 585)	560 (365, 680)	0.003
**ART**	75%	77%	75%	0.95
**NRTIs**	70%	76%	71%	0.79
**NNRTIs**	35%	33%	29%	0.88
**PIs**	55%	43%	46%	0.59
**HAART**	65%	66%	61%	0.89
**PI & NRTI-HAART**	35%	34%	32%	0.98
**NNRTI and NRTI-HAART**	10%	23%	18%	0.45
**PI and NNRTI-HAART**	20%	9%	11%	0.32
**hsCRP** (μg/mL)	9.9 (0.5, 5.5)	1.6 (0.7, 3.2)	3.4 (1.7, 4.0)	0.020
**TNF-alpha** (pg/mL)	1.7 (0.9, 2.6)	1.5 (1.0, 2.1)	1.6 (0.8, 2.8)	0.88
**IL-6 (pg/mL)**	2.2 (1.0, 4.1)	1.5 (1.1, 2.5)	1.8 (1.4, 3.6)	0.077
**sICAM1-CD54** (ng/dL)	240 (189, 455)	270 (190, 374)	249 (172, 412)	0.99
**HOMA > 2.6**	16%	36%	63%	0.004
**CAC > 400**	11%	3%	0	0.18
**Common cIMT** (mm)	0.54 (0.51, 0.57)	0.59 (0.51, 0.65)	0.67 (0.57, 0.74)	0.005
**Abnormal Common cIMT** (> 0.645 mm)	16%	26%	58%	0.003

Normal distributed data presented as average ± SD.

Non-normal distributed data presented as median (min. and max)

**Table 5b:** Lipoprotein-associated parameters in patients, tertiled by BMI.

VARIABLE	BMI < 21 (n=20)	BMI 21–30 (n=128)	BMI > 30 (n=28)	P
**LDL-C**	107 (69, 133)	108 (81, 137)	98 (84, 119)	0.82
**Triglycerides**	131 (90, 178)	125 (78, 207)	138 (87, 250)	0.47
**RLP-C**	9.7 (6.4, 14.7)	8.8 (6.5, 15.6)	8.1 (5.9, 21.6)	0.75
**NEFA (**mEq/L)	0.3 (0.2, 0.5)	0.3 (0.2, 0.5)	0.4 (0.3, 0.5)	0.076
**Apo-B**	81 ± 22	85 ± 22	84 ± 19	0.72
**Lp(a)**	8 (1, 27)	19 (8, 52)	14 (6, 29)	0.046
**HDL-C**	34 (27, 50)	37 (30, 48)	37 (27, 46)	0.94
**HDL-TG**	23 (14, 26)	17 (11, 21)	18 (12, 26)	0.27
**ApoA-I**	121 ± 26	126 ± 28	126 ± 30	0.67
**ApoA-II**	36 ± 10	35 ± 10	32 ± 8	0.36
**ApoE**	4.3 (3.6, 5.5)	4.0 (3.4, 5.1)	4.3 (3.5, 6.9)	0.47
**Preβ-1**	32 (24, 43)	37 (30, 46)	36 (28, 46)	0.41
**Preβ-2**	1.7 (1.7, 2.2)	1.5 (1.0, 2.8)	2.1 (1.0, 3.6)	0.48
**α-1**	10 (6, 17)	10 (6, 16)	11(8, 20)	0.43
**α-2**	26 (23, 40)	28 (22, 35)	28 (23, 37)	0.96
**α-3**	22 (17, 29)	23 (18, 28)	21 (17, 25)	0.45
**α-4**	7 (6, 8)	7 (5, 9)	6 (5, 11)	0.97
**Preα-1**	2 (1, 4)	2 (1, 5)	3 (1, 8)	0.49
**Preα-2**	4 (2, 6)	3 (2, 4)	5 (2, 6)	0.22
**Preα-3**	3 (1, 4)	3 (2, 4)	3 (2, 4)	0.94
**Preα-4**	1.2 (0.6, 1.7)	1.1 (0.7, 1.5)	1.4 (0.6, 1.8)	0.39
**CETP** (μg/mL)	2 (2, 3)	2 (2, 3)	2 (2, 3)	0.99
**LCAT** (μg/mL)	7 (6, 9)	7 (6, 8)	7 (6, 8)	0.94
**CETP Activity** (μg/mL/hour)	0.9 (0.2, 2.4)	0.3 (−1.3, 1.8)	0.3 (−1.4, 1.8)	0.53
**LCAT Activity** (μg/mL/hour)	17 (11, 27)	17 (10, 28)	13 (11, 21)	0.59

Values are mg/dL or as indicated.

Normally distributed parameters are expressed as average ± SD.

Not normally distributed parameters are expressed as median (min, max).

## Abbreviations

ART: Antiretroviral therapy
Apo: Apolipoprotein
ABCS1: ATP binding cassette transporter A1
BMI: Body mass index
CVD: Cardiovascular disease
cIMT: Carotid Intimal Medial Thickness
CETP: Cholesterol ester transfer protein
CT: Computed tomography
CD: Costimulatory protein
CRP: C-reactive protein
HDL-C: High-density cholesterol
HAART: Highly active antiretroviral therapy
HOMA: Homeostatic model assessment
HIV: Human immunodeficiency virus
IL: Interlukin
IDL: Intermediate-density lipoprotein
LCAT: Lecithin-cholesterol acyltransferase
Lp(a): Lipoprotein(a)
LDL-C: Low-density lipoprotein cholesterol
MI: Myocardial infarction
NEFA: Non-esterified fatty acids
NNRTI: Non-nucleoside reverse transcriptase inhibitor
NRTI: Nucleoside reverse transcriptase inhibitor
PI: Protease inhibitor
RLP-C: Remnant lipoprotein cholesterol
SRBI: Scavenger receptor BI
sICAM: Soluble Intercellular Adhesion Molecule
TG: Triglyceride
TNF: Tumor Necrosis Factor
VLDL: Very low-density lipoprotein
